# Microbial Blends: Terminology Overview and Introduction of the Neologism “Skopobiota”

**DOI:** 10.3389/fmicb.2021.659592

**Published:** 2021-07-02

**Authors:** Giovanni Del Frari, Ricardo Boavida Ferreira

**Affiliations:** LEAF—Linking Landscape, Environment, Agriculture and Food—Research Center, Instituto Superior de Agronomia, Universidade de Lisboa, Lisbon, Portugal

**Keywords:** microbiome, microbial consortium, microbial inoculant, synthetic microbiome, synthetic community, microbial cocktail

## Introduction

Over the last 20 years, numerous definitions have been proposed for the term microbiome (Berg et al., 2020). One of the most cited, albeit not the earliest (Whipps et al., 1988), was that introduced by Joshua Lederberg, who referred to the microbiome as “the ecological community of commensal, symbiotic, and pathogenic microorganisms that literally share our body space […]” (Lederberg and Mccray, 2001). This definition became especially popular as, over the following years, its meaning shifted from the organisms as taxonomical units (i.e., microbiota) to their collective genetic material. Four years after Lederberg's publication, the word microbiome began to be employed in the scientific literature (Nicholson et al., 2005), and to date, it has been used in tens of thousands of scientific publications. However, as its popularity increased, numerous definitions for the word microbiome appeared in scientific literature, sparking a hot debate over the birth and evolution of the meaning of this word (Prescott, 2017; Morar and Bohannan, 2019; Berg et al., 2020).

Over the last decade, advancements in next-generation sequencing allowed for a new understanding of the microbial complexity tightly associated with living beings and environments. Recent research demonstrated that microbiomes play a key role in the health of the organisms whom they are associated with, influencing, among others, their physiology, biochemistry, and reproductive success (Dinan and Cryan, 2017; Bai et al., 2020; Compant et al., 2020). In fact, the disruption of microbial homeostasis leads to dysbiosis, a condition which plays a major role in disease in humans and other animals (Liu et al., 2020), and decline in plants (Bettenfeld et al., 2020). Undoubtedly, much research has yet to be carried out to further understand the composition, functions, and resilience to stressors of microbiomes (e.g., anthropic activities and climate change). However, in recent years, microbiome research has branched out into a new direction. The acquired awareness over the role that microbial communities play in the health of biological systems led to the search for strategies to exploit microbiomes and microbial blends to achieve specific goals, such as restoring compromised systems (e.g., reversing a condition of dysbiosis) or enhancing existing ones.

## Microbiomes and Microbial Blends

The employment of microbiomes to produce positive effects on individuals or environments has been recently proven possible. One of the most popular examples is that of fecal transplants in humans. This technique is currently used to treat recurrent *Clostridium difficile* infections, and it showed promising results in treating inflammatory bowel diseases (Vindigni and Surawicz, 2017). Microbiome transplants have been successfully tested also in plant protection. For example, leaf endophytes of healthy *Phyllostegia hirsuta* were transferred on *Phyllostegia kaalaensis* leaves, significantly reducing disease severity caused by *Neoerysiphe galeopsidis* (Zahn and Amend, 2017). In another study, a soil microbiome transplantation significantly decreased the disease incidence in the *Solanum lycopersicum*–*Ralstonia solanacearum* pathosystem (Wei et al., 2019). Despite these promising results, there are numerous limitations still preventing the full exploitation of this technology. Microbiomes are often composed of hundreds, if not thousands, of species of microorganisms, many of which have not yet been identified (e.g., uncultivable species) and many more are found in very low abundances (rare taxa). Moreover, the composition of microbiomes is known to vary in time and space, complicating the access to reliable and stable sources of specific microbiomes (Lawson et al., 2019; Berg et al., 2020).

Unlike microbiomes, microbial blends are intelligently designed to study their component behavior (e.g., ecological studies) or to carry out well-defined tasks (e.g., biotechnology and biological control). Most frequently, microbial blends are composed of a mixture of bacterial species or strains (Voges et al., 2019), although those based on fungal species (Del Frari et al., 2019) or a combination of fungi and bacteria (Shahab et al., 2018) are becoming increasingly popular. Contrary to microbiomes, microbial blends are exact and reproducible, originating from microorganisms cultivable *in vitro*. For this reason, when employing a microbial blend, the outcome of its predicted function has a much greater reproducibility when compared to that of microbiomes. However, presently, microbial blends are relatively simple mixtures often composed of a few species and cannot match the results such as those achieved by microbiome transplants. It is worth remembering that, despite the fact that microbial blends are engineered with the intent of containing exclusively specific microorganisms, under some circumstances, endohyphal bacteria (Arora and Riyaz-ul-hassan, 2018), bacteriophages, and/or mycoviruses may be unwillingly added to the final mixture.

The numerous advantages of using microbial blends over microbiomes or single strains have been attracting a growing interest in several fields of science (Compant et al., 2019; Santos et al., 2019). Among the numerous applications of microbial blends, we find probiotics (El Hage et al., 2019), biotechnology (Christiaens et al., 2019), bioremediation (Brune and Bayer, 2012), biocontrol (Del Frari et al., 2019), ecology (Voges et al., 2019), human disease (Pereira et al., 2020), crop enhancement (Allaga et al., 2020), and more. In addition, recent advances in microbial blend engineering (Lawson et al., 2019) suggest that this technology will strongly contribute to re-shape microbiological research in the near future.

## Are Microbial Blend-Related Terms Synonyms?

Over the last 10 years, a variety of terms have been employed to refer to microbial blends, some of which are often used interchangeably (Mabwi et al., 2021), namely:

(a) microbial consortium, also referred to as “bacterial consortium” or “fungal consortium,”
(b) microbial inoculant, also referred to as “bioinoculant,”
(c) synthetic microbial community, also referred to as synthetic community or synthetic microbial consortium,
(d) microbial cocktail,
(e) synthetic microbiome.

To assess the similarities and differences among these terms, we examined 90 recently published (2017–2021) peer-reviewed scientific articles (both original research and review articles), and we focused on four key characteristics:

1. Cultivability of microorganisms: All microorganisms used to produce the final blend originate from mother cultures cultivated *in vitro*.
2. Taxonomical identification of microorganisms: All microorganisms are identified at least down to species level. Alternatively, microorganisms identified at the genus level are given an isolated identification code (e.g., strain ID).
3. Microbial multiplicity: The microbial composition involves multiple microorganisms (two or more).
4. Blend reproducibility: The microbial composition, qualitative and quantitative, is exact and reproducible (e.g., based on colony-forming unit and optical density).

In addition, we summarily looked at the fields of science in which these terms were most frequently used.

The results of our literature survey are shown in Table 1, where, for ease of visualization, we report only a subsample of literature references.

**Table 1:** Terms frequently used to identify microbial blends and associated key characteristics.

	**Microorganism**		**Microbial blend**		**Literature subsample**
	**Culturable**	**Identified**	**Multiplicity**	**Reproducibile**	
Microbial consortium	+	+	+	+	Lee et al., 2018; Del Frari et al., 2019; El Hage et al., 2019; Colpa et al., 2020; Pereira et al., 2020; Dai et al., 2021
Microbial inoculant	+	+	–	+	Chen et al., 2018; Wang et al., 2018; Rafique et al., 2019; Rékási et al., 2019; Dong et al., 2020; Singh et al., 2020
Synthetic microbial community	+	+	+	+	Shahab et al., 2018; Carrión et al., 2019; Christiaens et al., 2019; Honjo et al., 2019; Wang et al., 2020; Krieger et al., 2021
Microbial cocktail	+	+	+	+	Magalhães da Veiga Moreira et al., 2017; Qu et al., 2018; Pasaribu et al., 2019; Pires et al., 2019; Tobin et al., 2020; Zheng et al., 2020
Synthetic microbiome	+	+	+	+	Venturelli et al., 2018; Timmis et al., 2019; Vandeplassche et al., 2019; Voges et al., 2019; Koskella, 2020; Mabwi et al., 2021

*The symbol “+” indicates the presence of the specific key characteristic, while the symbol “-” indicates its absence, in association with each term (refer to the text for the definition of the concepts of each key characteristic)*.

The literature survey revealed that four out of the five terms under examination, which are commonly employed to refer to microbial blends, share the four key characteristics of cultivability of microorganisms, taxonomical identification, microbial multiplicity and blend reproducibility. The only exception refers to the term microbial inoculant, which does not share, in all cases, the microbial multiplicity characteristic. Even though, in the last decade, microbial inoculants containing multiple microorganism species have been increasing in number, this term also refers to inoculants that contain a single microorganism species (Santos et al., 2019). In addition, the majority of studies that make use of this term, although not the entirety, are context specific, as it is used to indicate the addition of microorganisms to seeds or soil. All other terms have been employed in several fields of science. From our examination, some terms seem to be preferentially used in specific contexts—for example, the term microbial cocktail is often used in biotechnological research, while the term synthetic microbiome is frequently used in medical research. However, giving an accurate estimate of context specificity for each term is virtually unfeasible and certainly goes beyond the scope of this opinion article.

According to this analysis and in light of the fact that they are often used interchangeably, we conclude that the terms microbial consortium, synthetic microbial community, microbial cocktail, and synthetic microbiome can be considered synonyms. Nevertheless, we examined a relatively small number of recent papers (90); therefore, we recognize that, among the immense volume of literature where these terms are employed, exceptions may be occasionally encountered.

## Discussion

Scientific vocabulary is at the core of science, as it is our means to share abstract novel concepts and ideas with our peers. Therefore, its diversity must be considered a strength. However, in recent years, in the fields of applied microbiology, biotechnology, and microbial ecology, numerous terms have been employed to identify “a blend of multiple microorganisms engineered to carry out a specific function.” Microbial consortium, synthetic microbiome, microbial cocktail, and synthetic microbial community are some of the terms that normally refer to such a broad definition. Similarly to the word microbiome, which gained considerable popularity over the last 10 years, the immense potential represented by microbial blends suggests that the term chosen to refer to them may become as much popular in the near future. However, the presence of multiple terms, some of which we demonstrated to be synonyms, may complicate communication both within the scientific community and with the greater public.

Therefore, why should we consider introducing a new term?

(1) Simplification: The process of simplification in scientific terminology, which invariably leads to the coinage of neologisms, aims to reduce to a single word abstract ideas that can only be defined with multiple words (e.g., see Raad, 1989; Gibson et al., 2017). To date, all terms that refer to microbial blends are composed of two or three words. Therefore, creating a single-worded neologism will significantly simplify communication in the medium and long terms.
(2) A clear definition: A single-worded term is easier to use, provided that it is clearly defined. Within this definition, all four key characteristics associated with the current terms that refer to microbial blends must be addressed, limiting the room for individual interpretation.
(3) Set a standard: Offering a single-worded, well-defined term may discourage more synonyms from being coined and introduced in the scientific literature.

Within this context, we would like to introduce and propose, as a potential candidate, the word *skopobiota*, a neologism built from the Greek word *skopós*, which translates in “purpose.” The term *skopobiota* is defined as:

“A purposefully designed, exact, and reproducible blend of multiple species of taxonomically identified microorganisms, whose components may act additively or synergistically to accomplish a predefined function in or on a specific environment.”

This single-worded term puts emphasis on the purpose for which different microbial blends are engineered as well as its quantitative and qualitative composition, addressing all four key characteristics associated with microbial blends.

Our undertaking goes beyond facilitating communication within and beyond academia. As a famous quote by philosopher Ludwig Wittgenstein (1922) goes, “the limits of my language mean the limits of my world”, the limits of science depend on scientific terminology as well. From this perspective, the word skopobiota may have a significant impact on future microbiome and microbial blend research. It may shape scientific approaches and future terminology and prevent controversies (see the word microbiome, Berg et al., 2020). However, we recognize that our effort has its limits as we agree with Raad (1989), who stated that “perhaps more and more scientists are beginning to realize that inaccuracy is rooted in the nature of knowledge, as we uncover more of it, rather than in the words used to transmit that knowledge.”

To conclude, in our opinion, the introduction of novel terms in science tends to follow five main sequential steps. First, there must be a need to describe a novel abstract idea; second, such idea is shared with peers using current scientific vocabulary; third, simplification leads to the coinage of a single-worded term (neologism); fourth, such neologism is publicly proposed in scientific literature; and fifth, the proposed neologism may or may not gain popularity. With respect to microbial blends, we have just reached the fourth step, and only time and the best judgment of scientists will decide which direction scientific terminology shall take.

## Author Contributions

GDF conceived the idea and wrote the manuscript. RF revised the manuscript.

## Conflict of Interest

The authors declare that the research was conducted in the absence of any commercial or financial relationships that could be construed as a potential conflict of interest.
